# The Role of Autophagy in Childhood Central Nervous System Tumors

**DOI:** 10.1007/s11864-022-01015-6

**Published:** 2022-10-05

**Authors:** Yafeng Wang, Yiran Xu, Changlian Zhu

**Affiliations:** 1 Department of Hematology and Oncology, Henan Neurodevelopment Engineering Research Center for Children, Children’s Hospital Affiliated to Zhengzhou University, Henan Children’s Hospital，Zhengzhou Children’s Hospital, Zhengzhou, 450018 China; 2 Henan Key Laboratory of Child Brain injury and Henan Pediatric Clinical Research Center, Institute of Neuroscience and Third Affiliated Hospital of Zhengzhou University, Zhengzhou, 450052 China; 3 Commission Key Laboratory of Birth Defects Prevention，Henan Key Laboratory of Population Defects Prevention, Zhengzhou, China; 4 Center for Brain Repair and Rehabilitation, Institute of Neuroscience and Physiology, Sahlgrenska Academy, University of Gothenburg, 40530 Gothenburg, Sweden

**Keywords:** Autophagy, Children, Central nervous system tumors, Cell death, Chemosensitivity, Radiosensitivity

## Abstract

Autophagy is a physiological process that occurs in normal tissues. Under external environmental pressure or internal environmental changes, cells can digest part of their contents through autophagy in order to reduce metabolic pressure or remove damaged organelles. In cancer, autophagy plays a paradoxical role, acting as a tumor suppressor—by removing damaged organelles and inhibiting inflammation or by promoting genome stability and the tumor-adaptive responses—as a pro-survival mechanism to protect cells from stress. In this article, we review the autophagy-dependent mechanisms driving childhood central nervous system tumor cell death, malignancy invasion, chemosensitivity, and radiosensitivity. Autophagy inhibitors and inducers have been developed, and encouraging results have been achieved in autophagy modulation, suggesting that these might be potential therapeutic agents for the treatment of pediatric central nervous system (CNS) tumors.

## Introduction

Autophagy is a catabolic process that captures and degrades damaged proteins and organelles in lysosomes [1, 2]. It effectively degrades normal cell metabolites and helps to maintain the health of the body, and in some cases autophagy can selectively remove cellular components such as damaged or excess peroxisomes, endoplasmic reticulum, mitochondria, or DNA [3••] thereby reducing the accumulation of abnormal proteins and organelles and maintaining cell homeostasis. The role of autophagy in promoting survival and maintaining cell homeostasis has been widely confirmed at the cell and organ level. For example, selectively knocking out the *Atg5* or *Atg7* genes in the brain leads to the accumulation of polyubiquitinated proteins and neuronal degeneration in mice. The survival and proliferation of T cells depends on *Atg5* [4], and *Atg5*-deficient mice cannot survive during the neonatal period and their tissues show the presence of amino acid exhaustion and insufficient metabolism [5]. Finally, in the absence of growth factors, the autophagy of hematopoietic cells is significantly enhanced in order to maintain the ATP supply and cell survival.

Autophagy is a physiological process that occurs in normal tissues. When faced with external environmental pressures (e.g., amino acid deficiency, insufficient glucose supply, or reduced oxygen supply) or internal environmental changes (e.g., protein, DNA, or mitochondrial damage or microbial infection), cells can digest part of their contents through autophagy in order to resist metabolic pressure or to remove damaged organelles [6]. Basal levels of autophagy play an important role in maintaining homeostasis in normal tissues, while nutrient deficiency, hypoxia, DNA damage, and cytotoxicity can all induce increased levels of autophagy. The induction of autophagy can promote cell survival by adjusting the dynamic balance inside and outside the cell [7]; however, excessive autophagy can seriously affect embryonic differentiation and induce cell death. Therefore, changes in autophagy are closely related to various clinical diseases such as cancer, neurodegeneration, heart disease, liver and metabolic disorders, infectious diseases, and autoimmune diseases [8••, 9, 10]. In the past decade, autophagy has received more and more attention as a new target in the treatment of various diseases.

## Autophagy and tumors

Malignant tumors are an important cause of childhood death worldwide, and their incidence has increased in recent years. Although current anti-tumor therapies have led to a positive prognosis for many patients, the efficacy of these therapies is still limited for some tumors. Autophagy occurs frequently during tumorigenesis, and at different stages of tumor development autophagy may have opposite effects, such as being a tumor suppressor or a tumor-initiating factor. In general, autophagy protects cancer cells during chemotherapy, and this can easily lead to tumor resistance and the development of refractory cancers [11]. Many external stimuli can affect tumor autophagy, such as hypoxia, acidification of the tumor microenvironment, nutritional deficiencies, drug treatment, or infection [12]. In addition, tumor suppressors or oncogenes can regulate the autophagy pathway in cancer cells. For example, p53 status can change the role of autophagy in tumor progression [13, 14].

### Tumor-suppressing roles for autophagy

Autophagy was originally considered to be a tumor suppressor mechanism. This concept originated from early reports that the crucial autophagy gene *BECN1* is lost in 40–75% of human prostate, breast, and ovarian cancers [15, 16]. In genetic knockout mouse models of hereditary breast cancer, the deletion of *BECN1* promotes the activation of p53 and reduces the occurrence of tumors. However, the deletion of *BECN1* in human cancers and the deletion of the breast cancer 1 (*BRCA1*) gene cannot be disassociated, which indicates that *BECN1* is not a tumor suppressor in most human cancers. Thus, *BECN1* only exerts a tumor suppressor effect in genetic animal models of cancer [15].

**Fig. 1:**
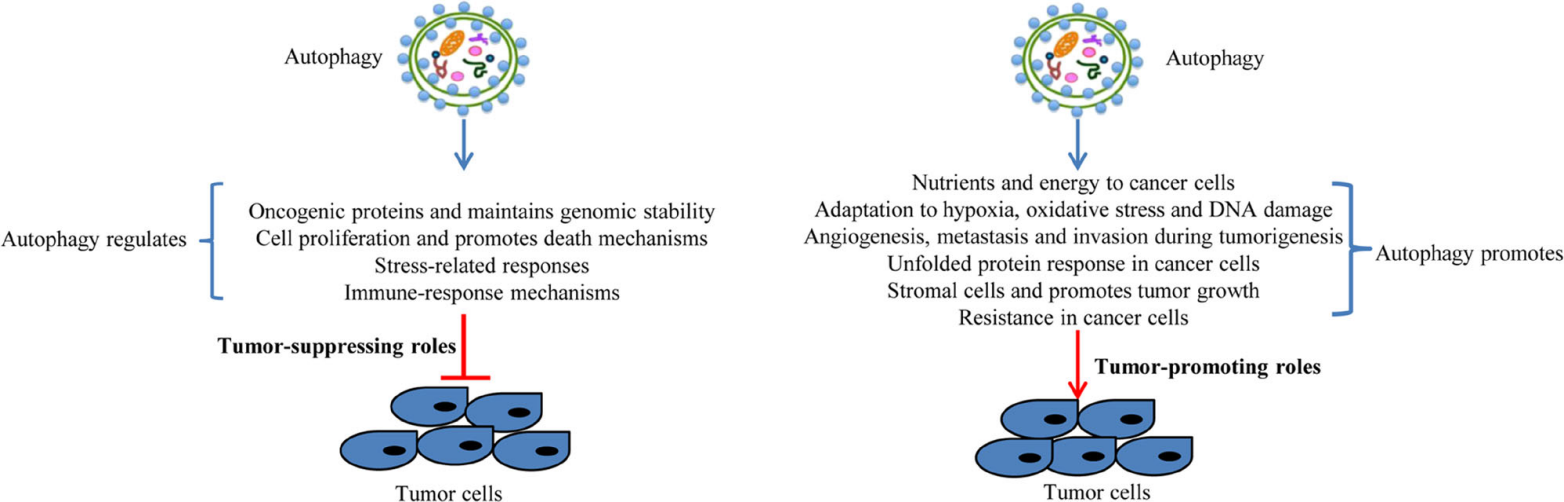
Tumor-suppressing and tumor-promoting roles for autophagy in cancer. The left panel shows the proposed mechanisms through which autophagy may suppress tumors by regulating oncogenic proteins, genomic stability, cell proliferation, cell death mechanisms, stress-related responses, and immune-response mechanisms. The right panel shows the proposed mechanisms for the tumor-promoting effect of autophagy by providing nutrients and energy to cancer cells, adaptation to oxidative stress and DNA damage, angiogenesis, metastasis and invasion during tumorigenesis, the unfolded protein response in cancer cells, tumor growth, and resistance to chemotherapy drugs in cancer cells.

The basal level of autophagy inhibits the occurrence of tumors by controlling the degradation of damaged components and proteins in cells [17]. Autophagy in genetic knockout mouse models can inhibit the accumulation of reactive oxygen species (ROS), DNA damage, tissue damage, inflammation, genome instability, and other tumor-initiating factors and thus can suppress tumors in their early stages [18]. Damage to mitochondria can lead to excessive ROS production, thereby promoting carcinogenesis, and autophagy prevents the occurrence of tumors by removing these malfunctioning mitochondria [19]. In mice lacking autophagy due to knockout of both *Atg5* and *Atg7*, oxidative stress and mitochondrial damage can induce hepatocytes to form liver tumors [20]. In genetic knockout mouse models of lung cancer, breast cancer, pancreatic cancer, and melanoma, deletion of the autophagy gene *Ras* or *Braf* inhibits the growth of benign tumors but accelerates the growth of malignant tumors. When exposed to chemical carcinogens, *Atg4*-deficient mice are more susceptible to fibrosarcoma [8••, 21]. Other studies have shown that the loss of autophagy-related genes such as *Atg3*, *Atg5*, and *Atg9* is also related to tumorigenesis [20]. Taken together, these results suggest that autophagy is an important mechanism for inhibiting tumor growth and that impaired autophagy can lead to tumor formation (Fig. 1, left panel).

### Tumor-promoting roles for autophagy

While basal levels of autophagy are low in normal cells and tissues, many cancer cell lines show high levels of autophagy [22•]. Autophagy promotes tumor growth and survival and the development of malignant tumors by maintaining the basic metabolic functions of tumor cells [23]. In addition, autophagy meets the metabolic needs of tumor cells by increasing stress tolerance and providing nutrients, and it maintains cell survival even under adverse conditions such as starvation or hypoxia, which are extremely common during tumor growth [18, 23]. Studies have confirmed that autophagy is upregulated in hypoxic tumor areas in the tumor microenvironment, thus inhibiting tumor-induced inflammation and promoting tumor cell survival [24]. Therefore, if tumor-promoting pathways are activated due to stress in tumor cells or to stress in the tumor microenvironment, this will increase the demand for autophagy, thereby promoting the growth and survival of tumors [25].

Studies have found that *RAS* mutations in tumor cells increase the level of autophagy, which can enhance tumor growth, survival, and deterioration, and such mutations are associated with the development of some cancers, including lung, colon, and pancreatic cancer [26–28]. Among the genetic knockout mouse models of lung cancer, pancreatic ductal adenocarcinoma, prostate cancer, and melanoma, tumors that were missing *Atg5* or *Atg7* grew slowly [29–31]. The deletion of *BECN1* in breast cancer cells and the deletion of *ATG13* or *ULK1* in glioblastoma cells led to similar results [32, 33]. Thus, inhibition of autophagy can induce tumor cell death.

Loss of *Atg5* or *Atg7* in mice can cause chronic liver damage, inflammation, and benign liver tumors, but these fail to develop into cancer [18]. As these tumors remain benign, this indicates that even though depletion of autophagy can increase tumor initiation in the livers of mice, autophagy is necessary for tumors to develop to the malignant stage [8••]. Specifically knocking out autophagy genes in mice can promote the formation of tissue damage and inflammation-related benign lesions, but many aggressive cancer cells require autophagy to grow and survive [34, 35]. The epithelial mesenchymal transition (EMT) plays an important role in tumor metastasis, and autophagy and EMT are interrelated [36, 37]. During tumor metastasis, cancer cells activated by EMT show high levels of autophagy and can survive in a variety of stressful conditions [38, 39]. Thus, autophagy can promote tumor development by promoting cancer cell proliferation and tumor growth (Fig. 1, right panel).

## The role of autophagy in childhood CNS tumors

CNS tumors are common solid tumors in children, with an average annual incidence of 1.7–4.1 per 100,000, which has increased slightly in recent decades and is second only to leukemia in terms of childhood tumors [40]. In recent years, despite great progress in early diagnosis, surgical procedures, and treatment strategies, the overall prognosis of CNS tumors is still very poor, with a 5-year survival rate of only 33% [41]. The histological analysis of childhood primary brain tumors and other CNS tumors in children between the ages of 0 and 14 years in the Central Brain Tumor Registry of the United States (CBTRUS) showed that the most common childhood CNS tumors are gliomas, medulloblastomas, atypical teratoid/rhabdoid tumors (ATRTs), craniopharyngiomas, and ependymomas. These are all malignant tumors, accounting for 80% of children’s CNS tumors [40, 42], and among all CNS tumors in children more than 90% are malignant [43].

Although childhood hematological tumors such as leukemia are still the main research focus for clinicians, CNS tumors have received more and more attention in recent years. The typical treatments of childhood CNS tumors, such as surgery, chemotherapy, and radiotherapy, have greatly improved the survival rate of children with CNS tumors. However, there are still some patients who respond poorly to treatment, and clinicians still need to find new therapeutic targets. Autophagy as a potential therapeutic target has been studied in a variety of cancers, which may provide a new therapeutic strategy for childhood CNS tumor patients. In this article, we focus on the most recent advances in the context of autophagy-dependent mechanisms driving childhood CNS tumor cell death, invasiveness, chemosensitivity, and radiosensitivity together with autophagy modulator treatment strategies that can be used to overcome autophagy-mediated drug resistance.

### Autophagy and cell death in childhood CNS tumors

Autophagy and cell death are two crucial cellular processes with complex protein networks, and there is a certain overlap in the regulatory mechanisms between them [44–47]. Studies have suggested that autophagy may serve as an alternative pathway to inducing cell death in many tumor cells with defects in apoptosis. For example, in BAX and BAK-deficient cancer cells endoplasmic reticulum stress-responsive apoptosis is prevented, but sustained autophagy can cause oxidative damage-induced cell death [44]. The autophagy and apoptosis signaling pathways are both separate and interconnected. Initially, autophagy and apoptosis were thought to differ as modes of cellular degradation in terms of morphology, biochemical indicators, molecules, and mechanisms. Later evidence showed that the two pathways can antagonize or promote each other under certain situations.

Autophagy was shown to be required for glioblastoma development in mice, and in light of the high resistance of malignant gliomas to apoptosis the induction of autophagic cell death by autophagy stimulators is an alternative method for triggering cell death in glioblastomas [48]. Temozolomide can induce apoptosis through the selective inhibition of autophagy, in which autophagic vehicles accumulate because their fusion with lysosomes is blocked. Modulation of the autophagic action of temozolomide with autophagy inhibitors can result in opposite outcomes depending on the step in the autophagic pathway that is targeted [49]. Su et al. identified a novel potential RAB13 inhibitor, which was confirmed to negatively regulate autophagy and induce cell death in low-grade glioma cells [50].

In other common type of childhood CNS tumors, the significant induction of autophagy produced by pimozide, a neuroleptic drug used for the treatment of schizophrenia and chronic psychosis, can promote medulloblastoma cell apoptosis by inhibiting the expression of the anti-apoptotic markers c-Myc, Mcl-1, and Bcl-2 [51]. In another in vitro study, plant-derived Δ9-tetrahydrocannabinol and cannabidiol induced cell cycle arrest in medulloblastoma and ependymoma cells in part through the production of ROS and the induction of autophagy and apoptosis [52].

Overall, these studies suggest a role for autophagy in regulating apoptosis in childhood CNS tumors. However, due to the complex network between autophagy and apoptosis, the effects of tumor–stroma interactions, and differences in the tumor microenvironment, more appropriate mouse models are required in order to clarify the function of autophagy in the apoptosis of CNS tumors in children.

### Autophagy and malignancy invasion in childhood CNS tumors

Childhood CNS tumors can acquire invasive properties by undergoing EMT, and this allows them to infiltrate into the surrounding normal brain tissue thus preventing complete surgical resection of the tumor. The autophagic process is closely related to tumorigenesis and the development of malignancies, and autophagy may have impacts on the invasiveness of brain tumors in children [53, 54].

Decreasing N-cadherin expression in glioblastoma cells has been shown to impair their focal adhesion and enhance their migratory capacity [55], and an in vitro study of glioblastoma autophagy has been shown to facilitate the degradation of SNAIL family proteins leading to upregulation of the N-cadherin level, which suggests that autophagy may suppress the invasive properties of glioblastomas [56, 57]. In addition, autophagy has also been reported to be involved in the regulation of the Wnt signaling pathway and the RKT Met signaling pathway, which are involved in the regulation of glioblastoma cell invasion through their effects on N-cadherin and vascular endothelial growth factor [58–60]. These findings demonstrated that autophagy can modulate different signaling pathways in glioblastoma cell lines, and it would be interesting to determine how this regulatory network can influence the invasion of glioblastoma cells. To investigate the effect of autophagy on the invasion of medulloblastoma, a recent study used shRNA-mediated knockdown of ATG5 in medulloblastoma cell lines belonging to the SHH group 3 and group 4 subtypes. Their findings showed that autophagy inhibition did not result in a significant difference in the proliferation or anchorage-independent growth of the medulloblastoma cells; however, autophagy inhibition led to a substantial reduction in the invasive potential of all three medulloblastoma cell lines [61]. In another study, the role of the pro-autophagy factor AMBRA1 in regulating medulloblastoma was identified showing that AMBRA1 expression depends on c-MYC levels and is correlated with poor prognosis in group 3 patients. Knockdown of AMBRA1 reduced the stemness, growth, and invasiveness of group 3 medulloblastoma stem cells [62]. Thus, it appears that regulation of autophagy profoundly affects the invasive potential and growth of childhood CNS tumor cells, which suggests a therapeutic potential for autophagy modulators in the treatment of pediatric brain tumors.

### Autophagy and chemosensitivity in childhood CNS tumors

Chemotherapy is still the main treatment for pediatric brain tumors; however, the prognosis of chemotherapy treatment in some groups of high-risk patients remains dismal. Intrinsic or acquired chemoresistance to chemotherapy drugs is a major clinical obstacle to the treatment of childhood CNS tumor patients. Therefore, a better understanding of the molecular mechanisms underlying chemoresistance to chemotherapy drugs may lead to improved clinical outcomes in pediatric brain tumor patients.

Recent studies have shown that modulation of autophagy in response to chemotherapy drug treatment, such as temozolomide, may hold great promise for circumventing chemotherapeutic resistance and improving anticancer efficacy in brain tumor patients [63, 64]. Li and colleagues demonstrated that the sensitivity of glioblastoma cells to temozolomide was increased by miR-519a, which might be mediated through autophagy, and that miR-519a overexpression could induce autophagy by inhibiting the STAT3/Bcl-2 pathway [65]. In another study, the authors used siRNA to knock down the autophagy-related genes *ATG12* and *ATG7* and then pharmacologically induced or inhibited these genes using rapamycin or chloroquine, respectively, to test the effect of autophagy on chemosensitivity in pediatric ATRT cell lines (BT-16 and BT-12). They found that silencing *ATG12* and *ATG7* or exposing the cells to the autophagy inhibitor chloroquine could inhibit this increase in autophagy; however, the effect of autophagy on killing tumor cells was minimal [66]. A recent study reported an oncogenic role for the nucleoporin TPR (translocated promoter region, a nuclear basket protein) in regulating heat shock transcription factor 1 (HSF1) mRNA trafficking, maintaining MTORC1 activity to phosphorylate ULK1, and preventing macroautophagy/autophagy induction in ependymomas. The authors found that high expression of TPR was associated with increased HSF1 and HSPA/HSP70 expression in ependymoma patients and showed that MTOR inhibition by rapamycin therapeutically suppressed TPR expression and reduced tumor size in an ependymoma mouse xenograft model [67]. These studies included both in vivo and in vitro experiments, and the findings showed that chemosensitivity in childhood brain tumors could be regulated by modulating autophagy levels, and this may have clinical relevance in the future planning of therapeutic regimens for pediatric brain tumors. However, autophagy is a dynamic process with multiple steps involved in producing autophagosomes, fusing with lysosomes, and completing the degradation of intra-vesicular contents, and it is feasible that blocking autophagosome formation has different effects on tumor cell survival than blocking autophagic flux. More research is needed to elucidate the mechanisms by which autophagy modulates the different chemotherapeutic agents, but at least for now autophagy as a potential therapeutic target provides new strategies for the treatment of childhood CNS tumors.

### Autophagy and radiosensitivity in childhood CNS tumors

Due to the local growth patterns of childhood CNS tumors, complete surgical removal is difficult in many patients and postoperative radiotherapy is necessary. Recently, autophagy has been reported to be involved in the regulation of radiosensitivity in childhood CNS tumors in both in vivo and in vitro studies [68–70], which suggests a new treatment strategy for pediatric brain tumor patients who are sensitive to radiation therapy. In a recent study, the authors assessed FOXG1 expression in glioma tissues and glioma-adjacent tissues, and they found that the FOXG1 expression level was up-regulated in glioma cells following exposure to irradiation and that FOXG1 reduced the radiosensitivity of glioma cells by promoting autophagy [68]. Lee et al. assessed the therapeutic effects of combining disulfiram with radiation treatment in ATRT cells (SNU.ATRT-5 and SNU.ATRT-6) and showed that disulfiram enhanced the radiosensitivity of ATRT cells with a reduction in the survival fraction and increased DNA double-strand breaks, apoptosis, autophagy, and cell cycle arrest in irradiated ATRT cells [69]. These studies suggest that autophagy might be a novel modulator for those childhood CNS tumor patients who receive radiotherapy; however, the outcome of autophagy observed in brain tumors after radiotherapy is not straightforward. Although an association between autophagy and radiosensitization was demonstrated, the precise role of autophagy in relation to brain tumor cell death is hard to define and more comprehensive analyses are needed.

### Autophagy modulators in childhood CNS tumors

Evidence for the effects of pharmaceutical modulators of autophagy in pediatric brain tumors is limited; however, the antitumor effect of autophagy remains an exciting potential treatment strategy. The *BRAF* (V600E) mutation is important in childhood CNS tumors [71], and a study showed that the autophagy inhibitor chloroquine reduced tumor viability in glioma cells with the *BRAF* (V600E) mutation [72]. The authors also demonstrated that chloroquine could improve vemurafenib sensitivity in children with ganglioglioma, indicating that pediatric CNS tumors with *BRAF* (V600E) are autophagy-dependent and should be targeted with autophagy inhibition in combination with other therapeutic strategies. Another autophagy modulator, salinomycin, can induce ROS in abortive autophagy, and this leads to regulated necrosis in glioblastoma cells [73]. For medulloblastoma, MirR-30a inhibited autophagy by reducing beclin 1/ATG5 expression and was linked to increased cell death in a medulloblastoma cell line [74]. Until now, there have only been a few clinical trials for autophagy modulators in treatment of childhood CNS tumors [75] (Table 1). Hydroxychloroquine combined with Dabrafenib (NCT04201457), everolimus (NCT00187174), everolimus combined with lenvatinib (NCT03245151), and Temsirolimus combined with valproic acid (NCT01204450) were used in clinical trials as modulators of induced autophagy in the treatment of childhood CNS tumors [76–79]. These clinical trials provide further evidence for autophagy modulators as potential therapeutic agents for the treatment of childhood CNS tumors.

**Table 1:** Clinical trials using Autophagy modulators in childhood CNS tumors

Tumor type	Study Phase (ID of Clinical trials)	Autophagy modulator	Autophagy Modulation	Ref.
Low-Grade Glioma	I/II (NCT04201457)	Hydroxychloroquine+ Dabrafenib	Inhibition	[76]
CNS tumor	I (NCT00187174)	Everolimus	Induction	[77]
CNS tumor	I/II (NCT03245151)	Everolimus Lenvatinib	Induction	[78]
CNS tumor + Neuroblastoma	I (NCT01204450)	Temsirolimus + Valproic Acid	Induction	[79]

## Autophagy and CNS tumor treatment-related brain injury

At present, the treatment of pediatric CNS tumors is still mainly based on surgery, radiotherapy, and chemotherapy. In particular, the related complications after multiple radiotherapy and/or chemotherapy sessions are still crucial issues that cannot be ignored in the clinic.

Neurotoxic brain injury caused by some anti-tumor chemotherapy drugs occurs widely in the clinic, and this reduces the treatment effect and quality of life of patients, especially for growing and developing children with CNS tumors [80]. Severe neurotoxic responses often result in patients facing the dilemma of reducing the dose of chemotherapeutic drugs, while at the same time such responses negatively impact on the patient's mental and physical state and their quality of life. Autophagy can regulate the extent of neurotoxic brain injury caused by anti-tumor drugs. For example, the peripheral nervous system damage caused by bortezomib can also enhance the level of autophagy [81]. Bortezomib is a proteasome inhibitor that prevents the degradation of misfolded proteins in the nervous system, resulting in severe neurotoxicity. Bortezomib can also activate the transcription factor ATF4 and up-regulate the expression of LC3-II, thereby activating autophagy [82]. By enhancing autophagy, proteasome inhibition can degrade protein aggregates and interfere with the neurotoxicity caused by bortezomib [83].

Radiotherapy is one of the most effective tools in the treatment of pediatric CNS tumors. However, damage to normal brain tissue surrounding the tumor constitutes a major problem and is associated with adverse side effects, particularly in pediatric patients. Autophagy is essential for survival, differentiation, development, and homeostasis [46, 84, 85], but inappropriate activation of autophagy is directly involved in triggering the initiation of apoptotic or necrotic cell death [86]. In an *in vitro* study, neural stem cells were shown to be extremely sensitive to irradiation [87]. *Atg7* knockdown significantly decreased autophagy, thus increasing apoptosis levels in irradiated neural stem cells, suggesting that autophagy protects NSCs from radiation-induced apoptosis. This indicated that downregulating autophagy by selective Atg7 knockdown in NSCs enhances radiation-induced neural stem cell damage, thus suggesting an important protective role for autophagy in maintaining neurogenesis. However, in a recent in vivo study using 10-day-old selective *Atg7* knockout mice subjected to a single 6 Gy dose of whole-brain irradiation, cell death and proliferation, microglia activation, and inflammation were reduced compared to wild type mice in the acute phase after irradiation. Selective neural deletion of the *Atg7* gene reduced irradiation-induced cerebellar white matter injury in the juvenile mouse brain by ameliorating oligodendrocyte progenitor cell loss in the subacute phase after irradiation [88, 89]. Together, the conflicting roles played by autophagy in different experimental conditions led to different protective effects of autophagy on neural stem cells in the above mentioned in vivo and in vitro studies. But at least, these results suggest that autophagy might be a potential target for brain injury after radiotherapy in pediatric CNS tumor patients.

## Conclusion

Numerous autophagy inhibitors and inducers have been developed, and encouraging results have been achieved in autophagy modulation. Preclinical trials of autophagy inhibitors and inducers combined with chemotherapeutics or radiotherapy may improve their efficacy and their therapeutic effects in cancer patients; however, clinical trials focusing on autophagy control are limited to only a few autophagy promoters and inhibitors. Therefore, further research is needed to determine their anti-tumor efficacy, including autophagy modulators and chemotherapeutic drugs that are used for pediatric CNS tumor patients. In addition, more research must be conducted in order to develop specific therapeutic agents for the treatment of pediatric CNS tumors.
